# Thromboelastometric evaluation of coagulation profiles of cold-stored autologous whole blood: A prospective observational study

**DOI:** 10.1097/MD.0000000000017357

**Published:** 2019-09-27

**Authors:** Shihoko Iwata, Yuji Hirasaki, Minoru Nomura, Makoto Ozaki

**Affiliations:** a Department of Anesthesiology, Tokyo Women's Medical University Hospital; b Department of Anesthesia, IMS Tokyo-Katsushika General Hospital, Tokyo, Japan.

**Keywords:** autologous, blood transfusion, fibrinogen, thromboelastometry

## Abstract

Preoperative autologous blood donation is a well-established procedure to reduce the need for allogeneic blood transfusion. We hypothesized that coagulation activity is maintained in cold-stored whole blood, because the fundamental polymerization properties of fibrin are preserved.

Fifty adult patients who underwent elective cardiothoracic surgery were enrolled.

Autologous whole blood collected 2 to 4 times at almost 1-week intervals before surgery was stored at approximately 4°C until reinfusion at the time of surgery. Blood samples were drawn just before reinfusion, and rotational thromboelastometry variables and fibrinogen levels were measured.

A total of 158 samples were analyzed. The mean duration of cold storage was 16.7 ± 7.4 days (range: 6–33 days). Platelet counts were very low due to collection through a leukoreduction filter. The mean fibrinogen level was 2.3 ± 0.6 g/L. Amplitude at 10 minutes after CT (A10), amplitude at 20 minutes after CT (A20), and maximum clot firmness (MCF) values as determined by FIBTEM analysis were 10.8 ± 3.8, 12.2 ± 4.2, and 13.1 ± 4.7 mm, respectively. Fibrinogen levels were strongly correlated with A10, A20, and FIBTEM-MCF values (ρ = 0.83, *P* < .0001, ρ = 0.84, *P* < .0001, ρ = 0.85, *P* < .0001, respectively). Fibrinogen levels were not correlated with the duration of cold storage (ρ = 0.06, *P* = .43).

The results of the present study demonstrate that fibrin polymerization occurs in cold-stored autologous whole blood, and that such activity is strongly correlated with fibrinogen levels. Furthermore, our data suggest that cold-stored leukoreduced autologous whole blood retains fibrin polymerization properties throughout 33 days.

## 1 Introduction

Preoperative autologous whole blood donation is a well-established transfusion practice in elective surgeries.^[1]^ Although the cost effectiveness of autologous whole blood donation remains controversial,^[1–5]^ it has the benefit of reducing patient exposure to allogeneic blood transfusion, which has been associated with various complications, including transfusion-transmitted viral infection, acute lung injury, hemolytic transfusion reactions, anaphylaxis, and increased morbidity and mortality.^[6]^ The primary purpose of autologous whole blood transfusion is to preserve and replace red blood cells rather than coagulation factors. On the contrary, some studies have suggested that cold storage (4°C) with citrate, phosphate, dextrose, and adenine (CPD-A) anticoagulant solutions can preserve coagulation factors in whole blood for up to several weeks.^[7–9]^ However, few studies have focused on the coagulation profile of cold-stored autologous whole blood for clinical use. Our clinical experience has been that hemostasis was achieved more successfully than we anticipated after autologous transfusion during cardiac surgery. This prompted our investigation of the fibrin polymerization properties of cold stored autologous whole blood. Because the fundamental capacity of fibrinogen is preserved in cold-stored whole blood for several weeks,^[7–9]^ we hypothesized that coagulation activity would be maintained in cold-stored autologous whole blood throughout the shelf life of 35 days. To evaluate this hypothesis, we investigated the coagulation profiles of cold-stored autologous whole blood using rotational thromboelastometry (ROTEM; Tem Innovations GmbH, Munich, Germany). Similarly, we investigated all possible correlations between fibrinogen levels, ROTEM variables, and cold storage duration.

## 2 Methods

The present observational study was approved by the Tokyo Women's Medical University Ethics Committee (approval number: 2747-R), and written informed consent was obtained from all patients prior to their participation in the study. This study was registered at the University Hospital Medical Information Network clinical trials registry on March 13, 2013 (registration number: UMIN000010238). Fifty adult patients (age ≥20 years) who underwent elective cardiac surgery at our hospital were consecutively enrolled between October 2013 and November 2014. Patients taking warfarin or who declined to participate were excluded. Autologous whole blood collection was performed in accordance with the standard protocol of our institution: a total of either 300 or 400 mL of whole blood was collected from each patient using a commercially available blood bag (Sepacell Integra CA system, Asahi Kasei Medical Co., Tokyo, Japan) with an integrated leukoreduction filter and containing 56 mL of CPD-A solution. Each blood bag was designed for the collection of a single 400-mL donation of whole blood in which autologous whole blood can be preserved for 35 days. Depending on the condition of patient, whole blood was collected 2 to 4 times before cardiac surgery. Basically, autologous blood donations were scheduled almost weekly, resulting in a total of 800 to 1600 mL of autologous whole blood per patient (Table 1). This was stored at approximately 4°C until reinfusion during cardiac surgery. Blood samples for the present study were drawn from the blood storage bag at the time of reinfusion. Autologous whole blood was transfused after weaning from cardiopulmonary bypass or when arterial blood gas analysis showed that the hemoglobin level was lower than 70 g/L.

**Table 1:**
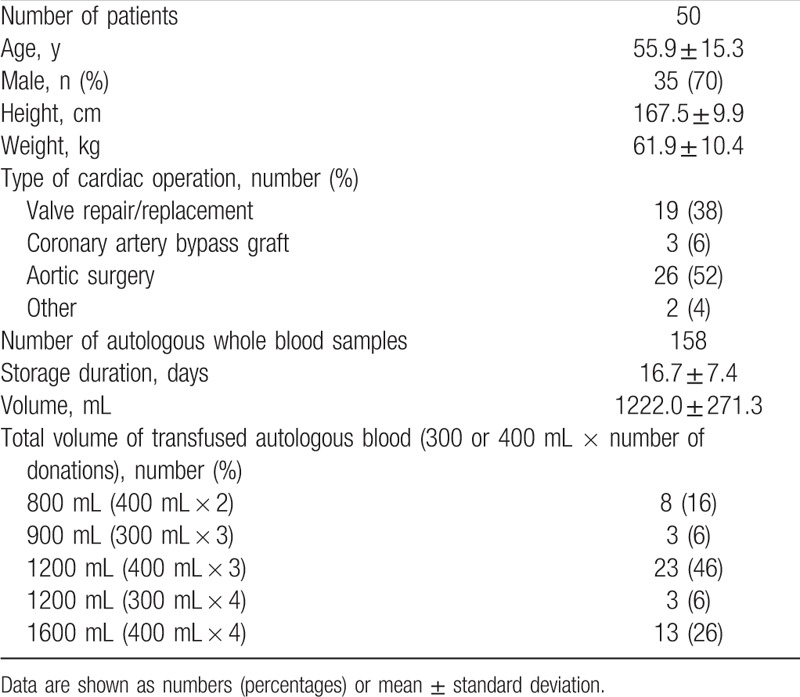
Patient characteristics, type of cardiac operation, and autologous whole blood data.

Standard hematological tests were used to assess the fibrinogen level (Clauss method, COAPRESTA 2000, Sekisui Medical Co., Ltd.; Shimadzu, Tokyo, Japan), leukocyte count, hemoglobin level (Hb), and platelet count (KX—21N Hematology Analyzer, Sysmex Co., Kobe, Japan) of each sample.

Because ROTEM analysis is sensitive to fibrinogen levels and platelet counts, it is commonly used to diagnose major causes of coagulopathy such as thrombocytopenia, hypofibrinogenemia, and hyperfibrinolysis.^[10–19]^ In the present study, we performed ROTEM analysis using the same blood samples from which standard hematological measurements were obtained. The ROTEM analysis consisted of INTEM (ellagic acid-activated coagulation profile), EXTEM (tissue factor-activated coagulation profile), and FIBTEM (EXTEM with platelet deactivation) tests. The following variables were assessed: clotting time (CT; s), clot formation time (CFT; s), alpha angle (α; degrees), amplitude at 10 minutes after CT (A10; mm), amplitude at 20 minutes after CT (A20; mm), maximum clot firmness (MCF; mm), and maximum lysis (ML; percentage decrease in amplitude relative to MCF achieved during measurement). The laboratory protocol is available at (DOI: dx.doi.org/10.17504/protocols.io.w46fgze).

For all parameters, normality of the data distribution was tested using the Shapiro-Wilk test. Values are presented as the mean ± standard deviation or median (interquartile range: 25th to 75th percentile). Associations between 2 continuous variables were assessed by linear regression analysis. Correlation of 2 continuous variables was assessed by calculating Spearman coefficient (ρ). Statistical analyses were performed using JMP Pro Statistical Software, Version 13.0.0 (SAS Institute Inc., Cary, NC). *P* values <.05 were considered statistically significant.

## 3 Results

Patient characteristics and clinical data are presented in Table 1. The mean volume of autologous whole blood collected from each patient prior to elective cardiac surgery was 1222.0 ± 271.3 mL (range: 800–1600 mL). The mean storage duration for all 158 autologous whole blood samples analyzed was 16.7 ± 7.4 days (range: 6–33 days) (Table 1). The mean Hb and fibrinogen levels were 106.1 ± 10.6 and 2.3 ± 0.6 g/L, respectively. Leucocyte and platelet counts were nearly zero in all samples (Table 2).

**Table 2:**
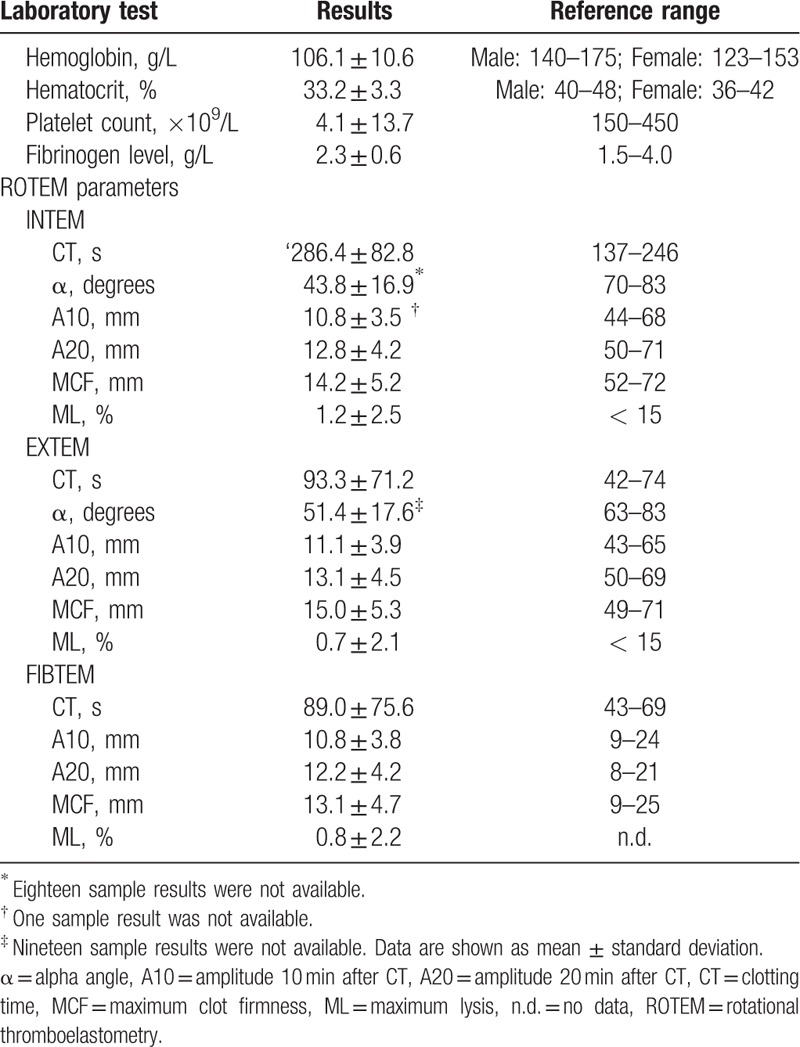
Results of standard blood tests and rotational thromboelastometry parameters.

Our analysis of INTEM, EXTEM, and FIBTEM results revealed that CT was prolonged significantly relative to reference standards (286.4 ± 82.8, 93.3 ± 71.2, and 89.0 ± 75.6 s, respectively). CFT was unmeasurable in almost all cases, because the amplitude of the samples did not reach 20 mm.

With regard to the INTEM α and EXTEM α values, 18 samples and 19 samples were unavailable, respectively, and almost all measured data were less than the reference values (Table 2).^[20]^ Moreover, INTEM and EXTEM values for A10, A20, and MCF were significantly below the respective reference values, although FIBTEM values for A10, A20, and MCF were all within the reference range (Table 2).

**Figure 1:**
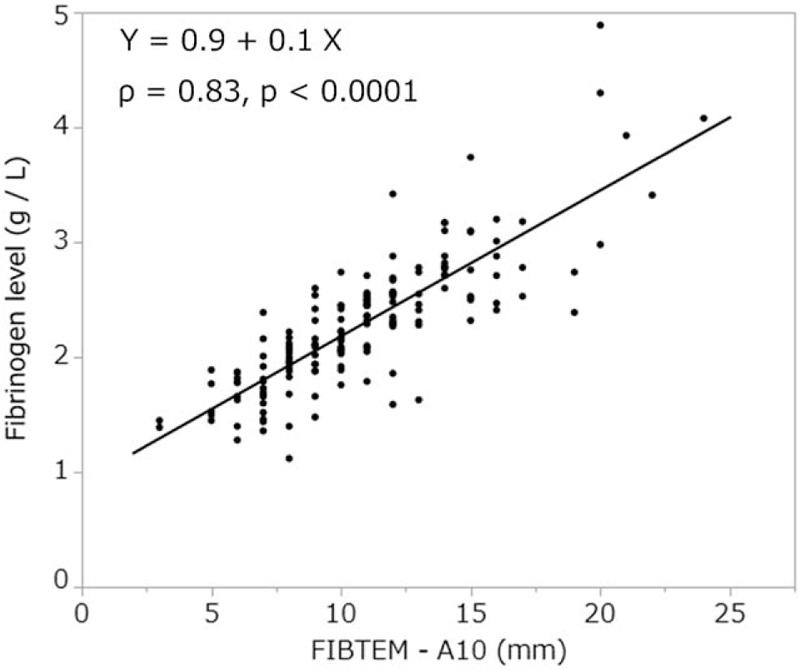
FIBTEM-A10 was strongly correlated with fibrinogen level. The FIBTEM-A10 test is commonly used for the clinical assessment of plasma fibrinogen level.

**Figure 2:**
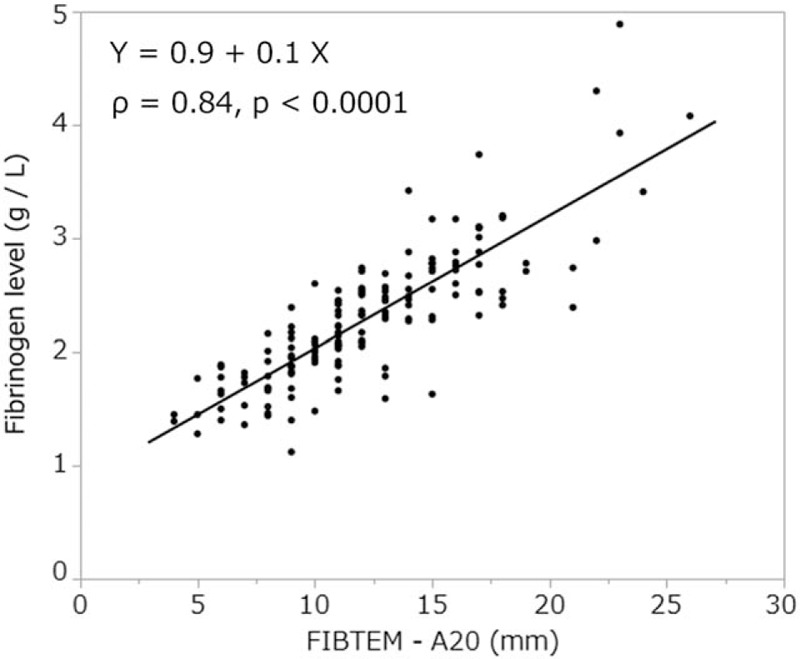
FIBTEM-A20 was strongly correlated with fibrinogen level.

**Figure 3:**
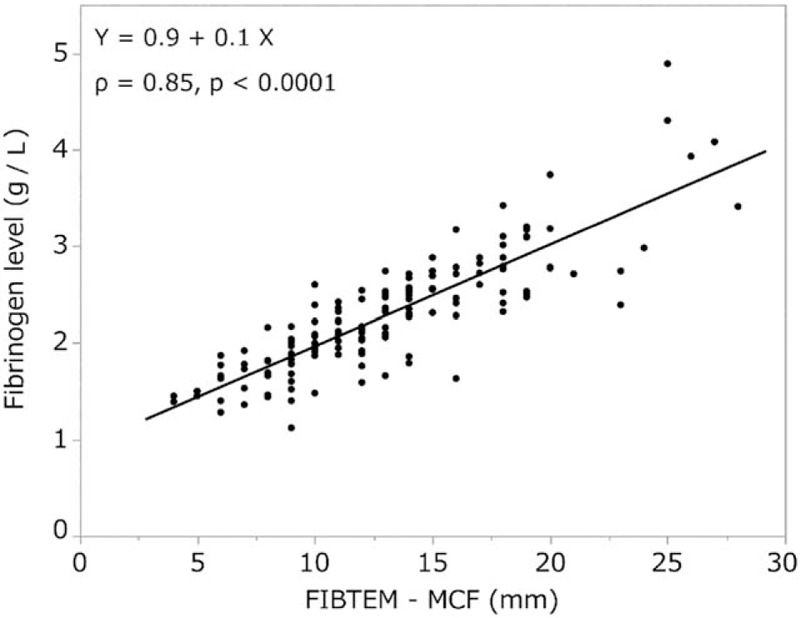
FIBTEM-MCF was strongly correlated with fibrinogen level. This close association indicates that the quantity and fibrin polymerization properties of autologous whole blood were retained for up to 33 days in cold storage. MCF = maximum clot firmness.

**Figure 4:**
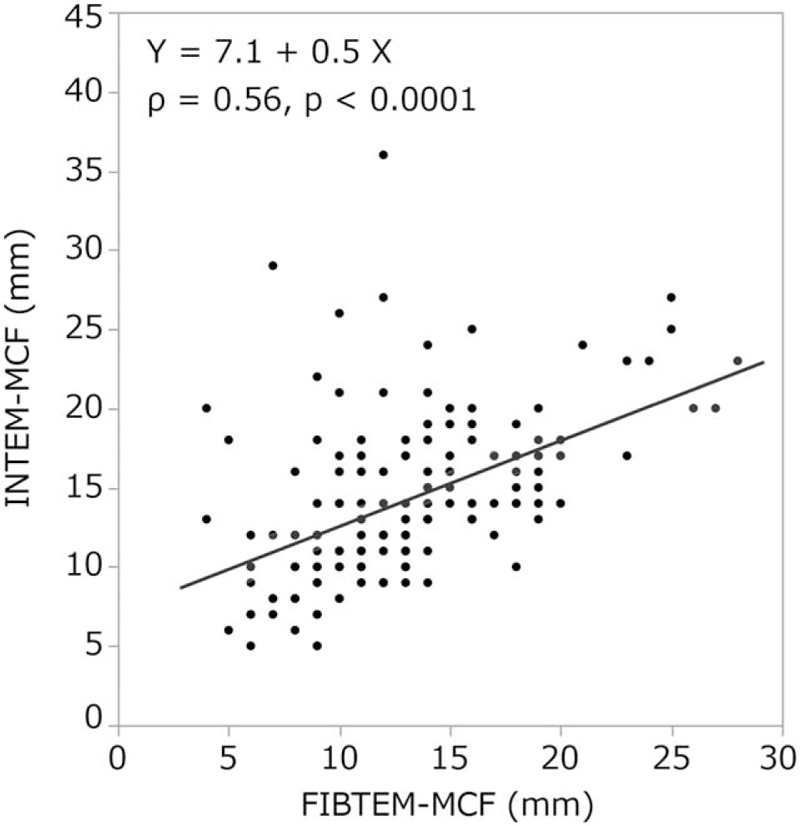
INTEM-MCF was moderately correlated with FIBTEM-MCF. MCF = maximum clot firmness.

**Figure 5:**
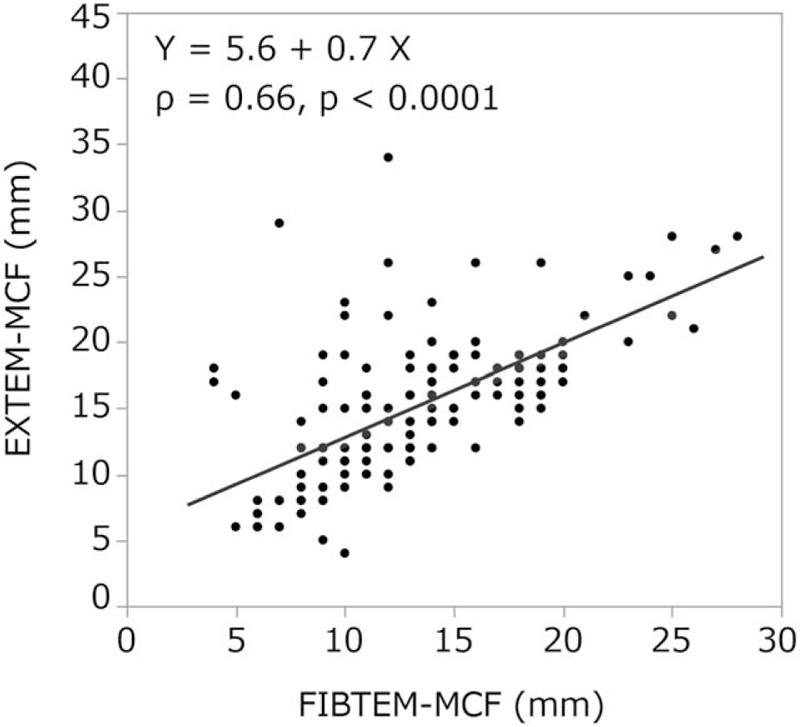
EXTEM-MCF was moderately correlated with FIBTEM-MCF. The MCF values of EXTEM and FIBTEM were very similar. The result could be explained by the deficiency in the platelet count. MCF = maximum clot firmness.

In the FIBTEM test, there was a strong correlation between A10, A20, MCF values, and fibrinogen levels [*ρ* = 0.83, *P* < .0001 (Fig. 1), *ρ* = 0.84, *P* < .0001 (Fig. 2), and *ρ* = 0.85, *P* < .0001 (Fig. 3), respectively]. In addition, FIBTEM-MCF was moderately correlated with INTEM-MCF (ρ = 0.56, *P* < .0001, Fig. 4) and EXTEM-MCF (ρ = 0.66, *P* < .0001, Fig. 5).

**Figure 6:**
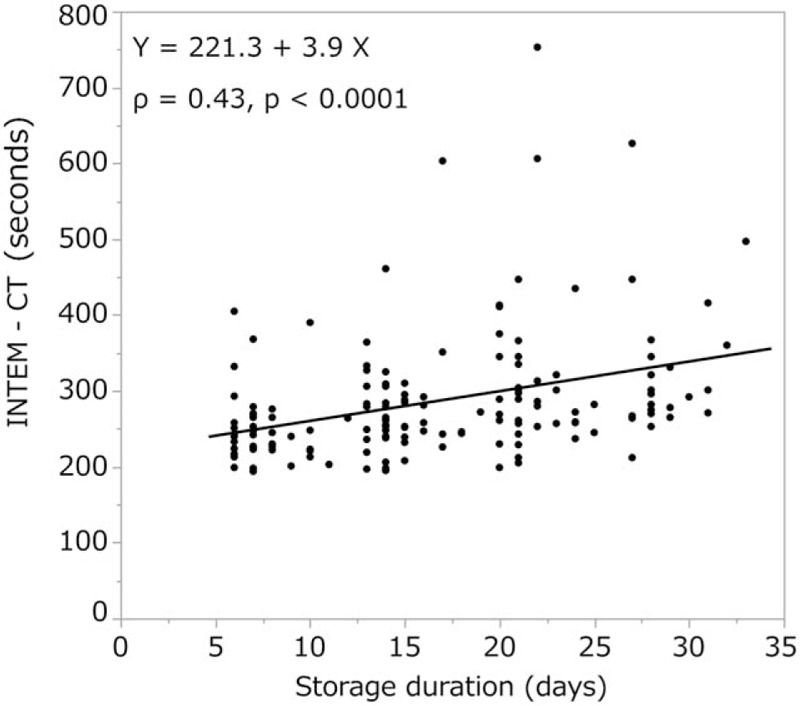
We observed a moderate correlation between storage duration and INTEM clotting time. CT = clotting time.

**Figure 7:**
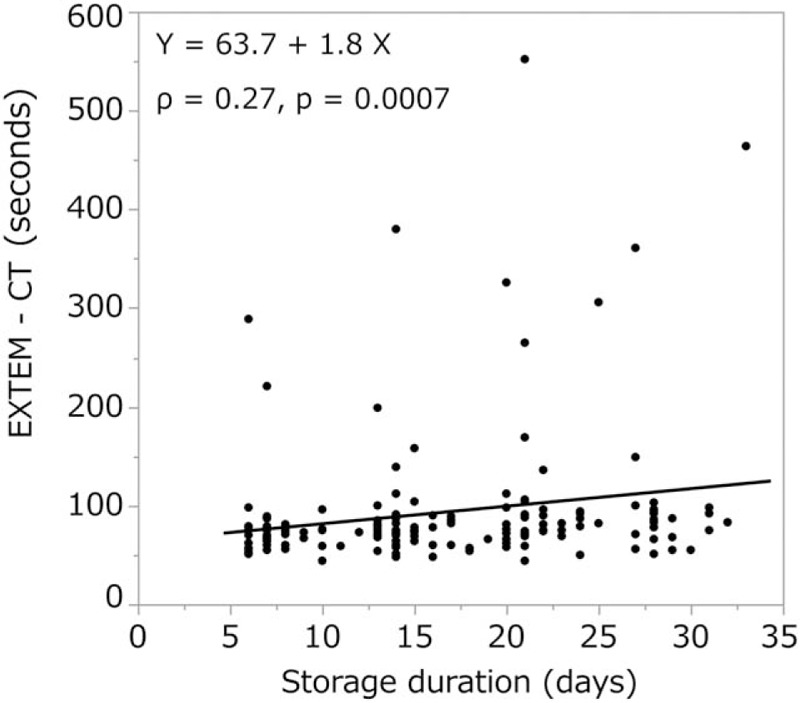
We observed a weak correlation between EXTEM clotting time in and storage duration. This may indicate that the level of extrinsic coagulation factors decreases with increasing cold storage duration. CT = clotting time.

**Figure 8:**
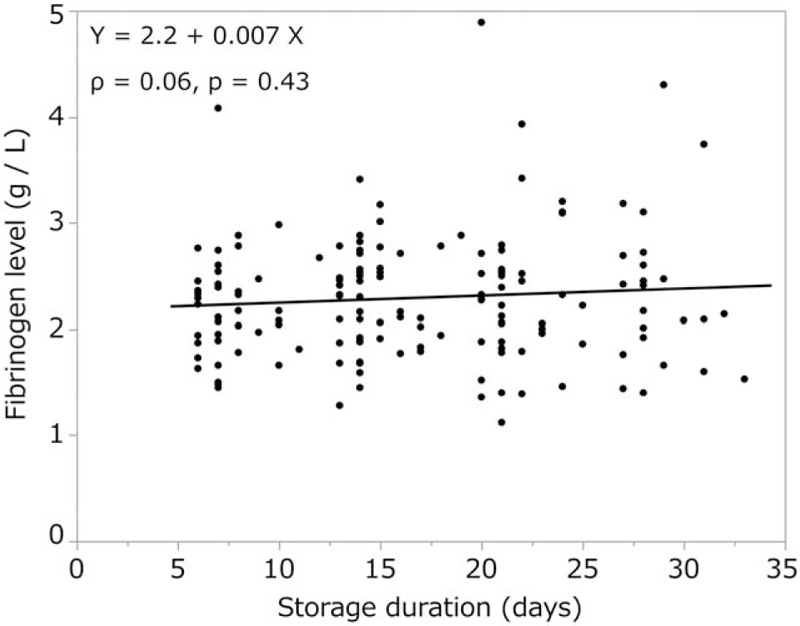
Storage duration was not correlated with fibrinogen level.

We observed a moderate correlation between storage duration and INTEM clotting time (ρ = 0.43, *P* < .0001, Fig. 6), and a weak correlation between storage duration and CT in the EXTEM (ρ = 0.27, *P* = .0007, Fig. 7) and FIBTEM tests (ρ = 0.33, *P* < .0001). The other ROTEM parameters (A10, A20, MCF in INTEM, EXTEM, and FIBTEM) or standard blood variables [fibrinogen level (Fig. 8), Hb, and hematocrit] showed no significant correlation with storage duration. ML values for INTEM, EXTEM, and FIBTEM analyses were 1.2 ± 2.5, 0.7 ± 2.1, and 0.8% ± 2.2%, respectively. As ML values >15% are indicative of progressive fibrinolysis,^[10–14]^ these results suggest that fibrinolysis was restricted in cold-stored autologous whole blood (Table 2).

## 4 Discussion

We aimed to determine whether the coagulation properties of cold-stored autologous whole blood are maintained until reinfusion, even if platelets are nearly eliminated by a leukoreduction filter. The ROTEM analysis is clinically used to determine viscoelasticity (amplitude and clot firmness) and is especially sensitive to fibrinogen levels and platelet counts. The FIBTEM test is the same as the EXTEM test but with the addition of cytochalasin D to prevent platelets from contributing to clot strength. The FIBTEM test is therefore commonly used for the clinical assessment of plasma fibrinogen. FIBTEM values for A10, A20, and especially MCF are strongly correlated with fibrinogen levels.^[10–12,15–19,21]^ Our data showed that the mean FIBTEM-MCF value was 13.1 ± 4.7 mm and the mean fibrinogen level was 2.3 ± 0.6 g/L, both of which were within the reference ranges (9–25 mm and 1.5–4.0 g/L, respectively). Moreover, a strong correlation between fibrinogen levels and FIBTEM-A10, A20, and MCF values was observed (ρ = 0.83, *P* < .0001, ρ = 0.84, *P* < .0001, and ρ = 0.85, *P* < .0001, respectively), and fibrinogen levels were not significantly correlated with storage duration (ρ = 0.06, *P* < .43). In addition, our data showed that the platelet count was nearly zero in all samples, which is likely due to the use of the integrated leukoreduction filter, which eliminates 99.9% of both leucocytes and platelets.

Moreover, our ML values during INTEM, EXTEM, and FIBTEM tests (1.2 ± 2.5, 0.7 ± 2.1, 0.8% ± 2.2%, respectively) remained <15%, indicating that fibrinolysis was not induced during cold storage. These results confirmed that clot strength was very little influenced by platelets and mainly depended on fibrinogen, which retains its polymerization properties and quantity despite being in cold storage for more than a month.

In ROTEM analysis, CT represents initial thrombin and fibrin formation. In cases in which EXTEM CT is >100 s and/or INTEM CT is >240 s, a thromboelastometry-guided transfusion algorithm in clinical practice recommends administration of fresh frozen plasma and/or a prothrombin complex concentrate because of the deficiency of coagulation factors.^[10,11,18,19]^ Notably, prolongation of EXTEM CT is associated with decreases or deficiency in the concentration of vitamin K–dependent coagulation factors^[11,18,19,22]^ or with the effects of anticoagulation treatment with vitamin K antagonist drugs; EXTEM CT ≥84 s predicts an international normalized ratio >1.5.^[23]^

In our study, both the INTEM CT (286.4 ± 82.8 s) and EXTEM CT (93.3 ± 71.2 s) were significantly longer than the reference ranges (137–246 and 42–74 s, respectively). Furthermore, CT in the INTEM test correlated moderately (ρ = 0.43, *P* < .0001) and CT in the EXTEM test correlated weakly (ρ = 0.27, *P* = .0007) with the duration of cold storage (Figs. 6 and 7). Thus, our findings suggest that the CT of autologous whole blood may increase as the duration of cold storage increases due to decreases in levels of coagulation factors other than fibrinogen.

For other ROTEM parameters, both CFT and α demonstrate the initial rate of fibrin polymerization. CFT represents the time necessary to achieve a clot firmness from 2 to 20 mm, and MCF is a measure of the maximal viscoelastic strength of the clot and depends on the firm aggregation of platelets and the formation of a stable fibrin network. A10 and A20 represent clot amplitudes 10 and 20 minutes after the onset of clot formation, respectively.^[13,14,20]^ INTEM and EXTEM values for CFT, α, A10, and MCF are significantly influenced by platelet counts, fibrinogen levels,^[10,15,16]^ and levels of coagulation factor VIII.^[17]^ Our data showed that CFT was unmeasurable in most cases, and α values in the INTEM and EXTEM tests were less than the reference values or unavailable. Moreover, INTEM and EXTEM values for A10, A20, and MCF were significantly below the reference values. The MCF of ROTEM represents fibrin polymerization and clot stabilization.^[17]^ In our data, INTEM-MCF and EXTEM-MCF were moderately correlated with FIBTEM-MCF (ρ = 0.56, *P* < .0001 and ρ = 0.66, *P* < .0001, respectively). In addition, the MCF values of INTEM, EXTEM, and FIBTEM were very similar. These results could be explained by the deficiency in the platelet count.

In the present study, the mean Hb level was 106.1 ± 10.6 g/L, although the mean Hb level measured in all patients just before autologous whole blood donation was 128.3 ± 11.1 g/L. The decreased Hb levels may have been due to dilution by the CPD-A anticoagulant solution, hemolysis, and/or the influence of in-line leukoreduction filtration or prolonged filtration.^[24]^

The use of a leukoreduction filter during collection of autologous whole blood is controversial. While there is no evidence that leukodepletion of preoperatively donated autologous whole blood improves patient outcomes,^[25,26]^ transfusion reactions are reported in 0.16% of patients who receive autologous whole blood.^[27]^ Although the mechanisms underlying these reactions remain unclear, previous studies suggest that storage-induced changes, such as the accumulation of platelet-derived cytokines, are responsible^[28]^ rather than leucocyte activity.^[29]^

In our hospital, a leukoreduction filter is used during autologous whole blood donation in order to minimize the risk of venous thromboembolism due to aggregate formation within the blood bag. Moreover, use of a leukoreduction filter is in accordance with the Japanese Society of Autologous Blood Transfusion guidelines.^[30]^

Our data showed that for up to 33 days, cold-stored leukoreduced autologous whole blood contained fibrinogen levels within the reference range and retained its fibrin polymerization properties. However, the clinical utility of this finding for hemostasis remains uncertain, because our ROTEM results show that although there is clot formation, it is slow and relatively weak in the absence of additional coagulation factors and platelets. Ranucci and Baryshnikova^[21]^ reported that a dose of fibrinogen concentrate <2 g might be sufficient to prevent bleeding during cardiac surgery. On the basis of this study and our own results, we calculated the desired volume of preoperative donated autologous whole blood as shown below assuming that 2 g of fibrinogen is needed in autologous whole blood, where autologous whole blood contains fibrinogen levels of 2.3 g/L and a hematocrit of 30%.

Desired volume of preoperative donated autologous whole blood 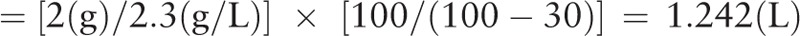


This exercise suggests that in terms of fibrinogen supplementation, at least 1.242 L of autologous whole blood may be needed to achieve a clinically helpful hemostatic effect. However, donating such amounts of blood may not be feasible before surgery. Furthermore, it is difficult to predict who will develop a major bleeding episode. In our study, several patients required a large amount of blood products in addition to the cold-stored autologous whole blood due to massive hemorrhage after cardiopulmonary bypass.

The present study possesses several noteworthy limitations. First, our analysis was limited by the small sample size. Second, we did not measure levels of other coagulation factors in the present study, which may have prolonged the CTs. Third, Hb levels, fibrinogen levels, and ROTEM parameters were not measured during the collection of autologous whole blood; therefore, we could not investigate changes in these parameters over time. Finally, because this study is based solely on in vitro data, the actual coagulation efficacy of autologous whole blood in hemorrhaging patients is not clear. Our study highlights the need for a prospective randomized trial to verify the clinical efficacy of autologous whole blood for hemostasis in surgical situations. However, despite this shortcoming, our results highlight the importance of future clinical studies comparing the in vivo properties of stored autologous and allogeneic blood infusion during and after surgery.

In conclusion, the results of the present study demonstrate that fibrin polymerization occurs in cold-stored autologous whole blood, and that such activity is strongly correlated with fibrinogen levels. Prolonged CTs are likely indicative of a loss of coagulation factors other than fibrinogen. Furthermore, our data suggest that cold-stored leukoreduced autologous whole blood retains fibrin polymerization properties throughout 33 days. However, it remains unclear whether the hemostatic effect of autologous whole blood would be sufficient in clinical settings. Further studies should focus on the effect of autologous blood donation on the perioperative blood coagulation profiles.

## Acknowledgments

The authors sincerely acknowledge Ms. Yoshimi Sugino and Ms. Toshiyo Kaneko for their excellent technical assistance. They would like to thank Editage (www.editage.jp) for English language editing.

## Author contributions

**Conceptualization:** Minoru Nomura, Makoto Ozaki.

**Data curation:** Shihoko Iwata.

**Formal analysis:** Shihoko Iwata.

**Funding acquisition:** Minoru Nomura, Makoto Ozaki.

**Investigation:** Shihoko Iwata.

**Methodology:** Shihoko Iwata, Yuji Hirasaki.

**Project administration:** Shihoko Iwata.

**Resources:** Shihoko Iwata.

**Writing – original draft:** Shihoko Iwata.

**Writing – review and editing:** Shihoko Iwata, Yuji Hirasaki.

Shihoko Iwata orcid: 0000-0002-4834-0405.
